# Impact of selected personal factors on seasonal variability of recreationist weather perceptions and preferences in Warsaw (Poland)

**DOI:** 10.1007/s00484-016-1220-1

**Published:** 2016-08-08

**Authors:** Katarzyna Lindner-Cendrowska, Krzysztof Błażejczyk

**Affiliations:** Department of Climatology, Faculty of Geography and Regional Studies, University of Warsaw, Warsaw, Poland

**Keywords:** Outdoor thermal comfort, Weather perception, Urban tourism, PET index, Warsaw

## Abstract

Weather and climate are important natural resources for tourism and recreation, although sometimes they can make outdoor leisure activities less satisfying or even impossible. The aim of this work was to determine weather perception seasonal variability of people staying outdoors in urban environment for tourism and recreation, as well as to determine if personal factors influence estimation of recreationist actual biometeorological conditions and personal expectations towards weather elements. To investigate how human thermal sensations vary upon meteorological conditions typical for temperate climate, weather perception field researches were conducted in Warsaw (Poland) in all seasons. Urban recreationists’ preference for slightly warm thermal conditions, sunny, windless and cloudless weather, were identified as well as PET values considered to be optimal for sightseeing were defined between 27.3 and 31.7 °C. The results confirmed existence of phenomena called alliesthesia, which manifested in divergent thermal perception of comparable biometeorological conditions in transitional seasons. The results suggest that recreationist thermal sensations differed from other interviewees’ responses and were affected not only by physiological processes but they were also conditioned by psychological factors (i.e. attitude, expectations). Significant impact of respondents’ place of origin and its climate on creating thermal sensations and preferences was observed. Sex and age influence thermal preferences, whereas state of acclimatization is related with thermal sensations to some point.

**Electronic supplementary material:**

The online version of this article (10.1007/s00484-016-1220-1) contains supplementary material, which is available to authorized users.

## Introduction

Over the last 15 years, many studies have been dedicated to investigate thermal comfort in outdoor environment and its relation to human thermal preferences. Chen and Ng (2012) as well as Kántor et al. (2012b) provided comprehensive comparative reviews of these approaches. The majority of weather perception studies have focused on differentiation of human thermal sensations and preferences in urbanized areas, such as various urban structures (Knez and Thorsson 2006; Eliasson et al. 2007; Bröde et al. 2012, Cohen et al. 2012, Kántor et al. 2012a, Krüger et al. 2013, Pearlmutter et al. 2014), urban parks (Thorsson et al. 2004, 2007; Knez and Thorsson 2008; Lin et al. 2013) or recreational areas (Oliveira and Andrade 2007). Most frequently, thermal perception studies have been conducted in relatively narrow thermal condition ranges, limited to the summertime (Pearlmutter et al. 2014; Saaroni et al. 2015), warm seasons (Thorsson et al. 2004; Kántor et al. 2012a; Krüger et al. 2013) or climatic zones characterized with small annual air temperature amplitude (i.e. tropical and subtropical climates) (Spagnolo and de Dear 2003; Krüger and Rossi 2011; Lin et al. 2011; Yin et al. 2012; Cohen et al. 2012). However, only a few all-year weather perception studies have been carried out in moderate climatic zone with large variations of thermal conditions during the year and with winter air temperature falling below 0 °C (Nikolopoulou and Lykoudis 2006; Eliasson et al. 2007; Lindner-Cendrowska 2013).

Human thermal sensation is a complex physiological, behavioural and psychological response to meteorological conditions. It is not only affected by physiological thermoregulation and clothing insulation but also by individual features, culture, mood and other social peculiarities (Humphreys 1995; Nikolopoulou and Steemers 2003). Although a lot of concern has been devoted to understand the impact of personal characteristics on thermal environment perception, it has not been finally settled how gender, age or health state influence human thermal sensations and preferences. It has been suggested that women have worst tolerance to cold weather conditions than men, due to their greater psychophysical sensitivity and characteristic clothing (dresses, skirts) which provides worse thermal insulation (Parsons 2002). They are also rather more susceptible to deviations from thermal optimum (Oliveira and Andrade 2007) and more critical of their thermal environment as they tend to feel too hot or too cold more often than males (Krüger and Rossi 2011). The impact of age on thermal preferences is less evident. It has been shown that in summer, older people feel warm and hot more frequently than younger ones, what can be associated with clothing insulation which becomes higher with age (Unger et al. 2008). Conversely, Krüger and Rossi (2011) have found younger respondents to be more sensitive to heat, while elderly less sensitive to variations of thermal conditions. At the same time, some investigators conclude that neither sex nor age have influence on human thermal sensations (Knez and Thorsson 2006; Bröde et al. 2012).

The significance of the influence of psychological factor on human weather perception has been emphasized in many publications (Nikolopoulou et al. 2001; Nikolopoulou and Steemers 2003; Spagnolo and de Dear 2003). People, while experiencing particular thermal conditions, confront them with their present knowledge and past experiences. This helps them to create individual ‘thermal memory’, which is a reference for their subjective assessment of outdoor thermal comfort and influences their expectations towards weather conditions in seasonal scale, but also from day to day (Höppe 2002; Nikolopoulou and Lykoudis 2006; Mansfeld et al. 2007; Lin et al. 2011). Moreover, thermal expectations greatly affect one’s satisfaction by the atmospheric environment and tolerance towards objectively less favourable meteorological conditions (Höppe 2002; Thorsson et al. 2004; Denstadli et al. 2011). In addition, people that keep positive attitude and stay outdoors out of their own desire tend to evaluate thermal conditions as more comfortable than they really are (Thorsson et al. 2004; Knez and Thorsson 2008; Lin 2009). The facultative participation in outdoor activities increases acceptability levels for a wide range of meteorological conditions (Nikolopoulou and Lykoudis 2006; Lin et al. 2013). It is thus suspected, that during voluntary activities, such as tourism and recreation, people, who gain pleasure and satisfaction from staying outdoors in particular thermal environment (even outside theoretically comfortable conditions), will perceive current weather in more positive and tolerant manner. In addition, as tourism is an international phenomenon, people of different origins have different cultural backgrounds and degrees of physiological adaptation to particular climate (Lin and Matzarakis 2008). Knez and Thorsson (2008) and Knez et al. (2009) have found that even under similar thermal conditions, different populations (in this context Japanese and Swedish) can vary in their evaluation of a given weather. Moreover, foreign visitors frequently tend to have different expectations and perception of the climate of tourist destinations than locals (De Freitas 2003). Usually, tourists do not have a ready definition of ‘good’ or ‘bad’ weather and their view of optimal meteorological conditions, as well as satisfaction from staying outdoors, may vary according to the origin of particular tourist and depending on holiday destination (Gómez-Martín 2005). This observation was confirmed by Scott et al. (2008) in tri-nation comparison study of climate preferences for various types of tourism.

The aim of this study was to assess thermal sensations and preferences of recreationists (i.e. tourists and people staying outdoors for recreational purposes) in urban environment in moderate climate zone, as well as to identify how personal factors (physical or physiological) modify bioclimate perception. To achieve this goal, weather perception surveys with concurrent micrometeorological measurements were conducted in the area of the Old Town of Warsaw (Poland). (1) The thermal sensations and preferences towards various meteorological elements were examined as well as (2) personal factors influencing thermal perception were identified.

## Materials and methods

### Study area

**Fig. 1:**
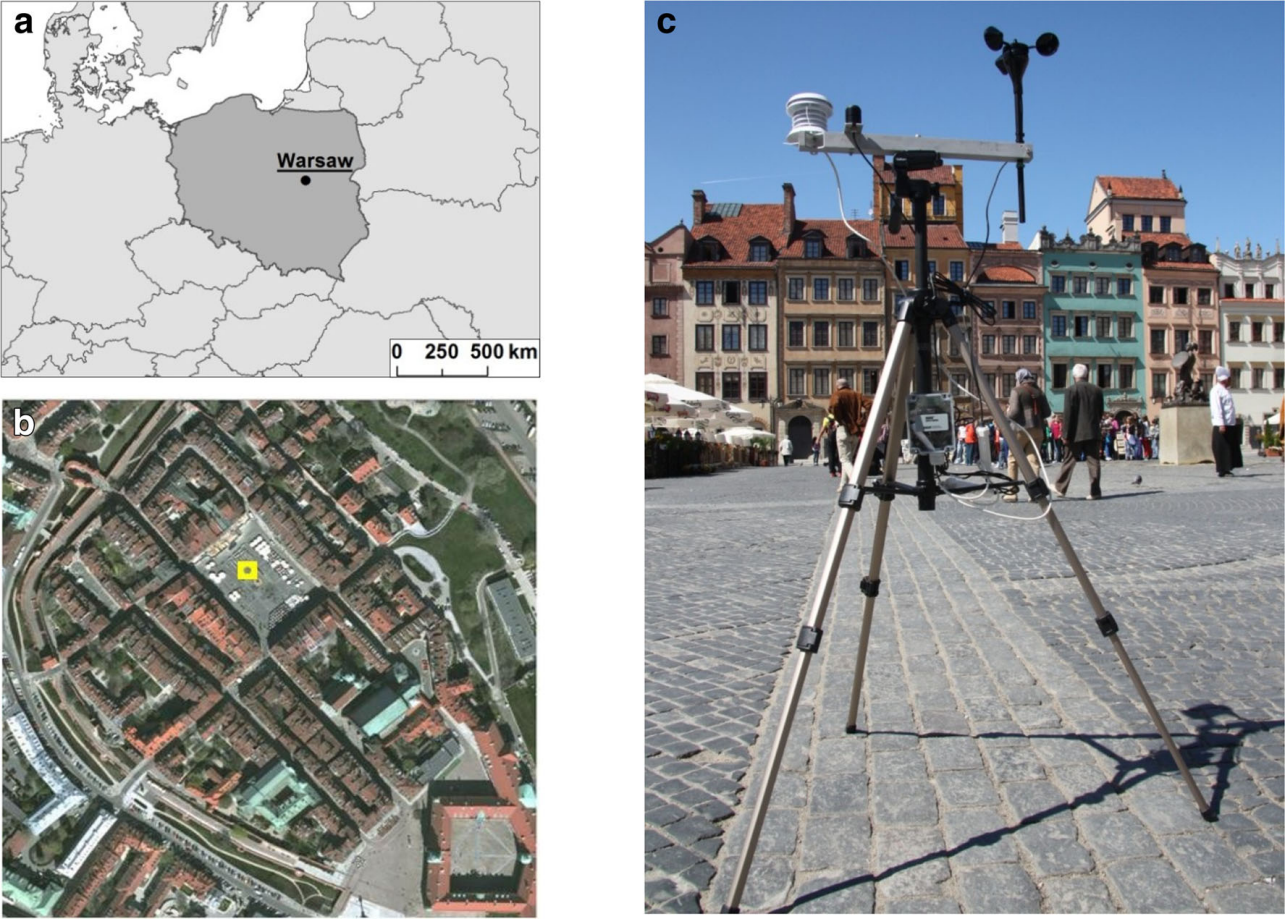
Geographic location of Warsaw (**a**) and the field study area in the Old Town of Warsaw (**b**). Weather station located on the Old Town in Warsaw (**c**)

Warsaw (52°14′N 21°1′E) is the capital and one of the most popular tourist destinations of Poland. As estimated, 7.5–8.3 million foreign and domestic visitors and tourists visited Warsaw in 2014 (Ipsos Loyalty 2015). The city is situated in central Poland in Middle-Mazovian Lowland (Fig. 1a). Its average elevation is around 100 m above sea level. According to Köppen-Geiger Climate Classification (Peel et al. 2007), climate of Warsaw is humid continental (Dfb) with cold, cloudy winter and warm summer. Mean annual air temperature is 8.5 °C (1981–2010) (*Institute of Meteorology and Water Management* 2015). Mean monthly temperature ranges between −1.9 °C in January and 19.0 °C in July. Maximum daytime temperature can exceed 30 °C from May to September, while minimum temperature below 0 °C can be registered from late September to May. Absolute maximum and minimum daily air temperature in Warsaw are 36.4 °C (1.08.1994) and −30.7 °C (8.01.1987), respectively. Yearly rainfall total is 531.5 mm and the wettest month is July (72.9 mm).

Field studies were conducted in the Old Town of Warsaw, which is the most popular district among tourists, located in the city centre, near the Vistula riverbank. Questionnaire surveys with accompanying micrometeorological measurements were carried out in the Old Town Marketplace, which is a rectangular (90 × 73 m) cobbled square, surrounded by three- or four-storey historic buildings (Fig. 1b).

To investigate seasonal variability of weather perception, four field study campaigns were conducted. The survey was carried out for several days in each season of the year on July 2010, February 2011, April 2011 and October 2011. The measurements and questionnaires took place between 11 a.m. and 4 p.m. (local time), when this area was mostly visited by recreationists and the whole place was well insolated. The study was carried out only on the days with no precipitation and in the spots unshaded by the buildings.

#### Micrometeorological measurements

Meteorological variables were measured using the HOBO® Micro Station (Onset Computer) which was equipped with air temperature and relative humidity sensor (S-THB-M008), cup anemometer (S-WSA-M00) and pyranometer (S-LIB-M003) (Fig. 1c). Air temperature (*Ta* in °C), relative humidity (*RH* in %) and global solar radiation (*Kglob* in W·m^−2^) were measured at 1.2 m above the ground. Wind speed (*v* in m·s^−1^) was measured at approximately 1.6 m height. Meteorological data from all sensors were scanned every 30 s and averaged after each 5 min.

The mean radiant temperature (*Tmrt* in °C) was calculated from *Ta*, *RH*, *v* and *Kglob* using BIOKLIMA software (Błażejczyk 2005). The formula applied was

$$ Tmrt={\left[\frac{\frac{R}{Irc}+0.5\cdot Lg+0.5\cdot La}{s_h\cdot \sigma}\right]}^{0.25}-273 $$

where *R*—absorbed solar radiation (W·m^−2^), *Irc*—the coefficient reducing convective and radiative heat transfer through clothing, *Lg*—ground radiation (W·m^−2^), *La*—atmosphere back radiation (W·m^−2^), *s*_*h*_—emissivity coefficient for humans (0.95) and *σ*—the Stefan-Boltzmann constant (5.667·10^−8^ W·m^−2^·K^−4^).

Absorbed solar radiation (*R*) was calculated from global solar radiation (*Kglob*) using the SolGlob model provided by BIOKLIMA. The equation forms varied according to solar elevation and *Kglob*/*Kt* (potential solar irradiation at clear sky) ratios. Detailed formulas are published elsewhere (Błażejczyk 2005) and (Błażejczyk and Kunert 2011).

Taking into account that one parameter alone is not sufficient for the assessment of thermal comfort conditions (Nikolopoulou and Lykoudis 2006), the measured *Ta*, *RH* and *v* as well as calculated in BIOKLIMA software *Tmrt* were used as input variables to calculate PET (°C, Physiological Equivalent Temperature) (Mayer and Höppe 1987; Höppe 1999). This index is a measure of human thermal sensations and is based on the MEMI human heat budget model (Munich Energy-balance Model for Individuals) (Höppe 1984). PET is defined as the air temperature at which, in a typical indoor environment (*v* = 0.1 m·s^−1^; *Ta* = *Tmrt*; *vp* = 12 hPa), the heat budget of the human body (dressed in clothing of 0.9 clo and performing light activity congruent with 80 W) is balanced with the same core and skin temperature which would occur under the assessed outdoor conditions. In order to calculate PET values, RayMan software was used (Matzarakis et al. 2007, 2010).

#### Questionnaire survey

Subjective assessments and preferences of the weather elements and thermal conditions were surveyed using a weather perception questionnaire, which was adapted from Spagnolo and de Dear (2003) as well as Oliveira and Andrade (2007) and designed in accordance with ISO 10551 (1995). The questionnaire consisted of three parts and its completion took 2 min on average. The survey was carried out either in Polish or in English and exact time of questionnaire was recorded, which subsequently enabled matching respondent answers to actual meteorological conditions. The first part of the questionnaire concerned thermal sensations and preferences towards meteorological elements (air temperature, humidity, wind speed, insolation and cloudiness). Seven-point thermal sensation ASHRAE scale was used to determine how respondents perceived thermal conditions, with −3 corresponding to ‘cold’, 0 to ‘neutral’ and +3 to ‘hot’ sensation. This type of scale has been used in earlier studies such as Thorsson et al. 2004, Lin 2009, Lin et al. 2011, Bröde et al. 2012 and Krüger et al. 2013. To investigate preferences towards weather elements, 3-point McIntyre’s scale was applied, with 0 meaning ‘no change’ desire and −1 or +1 meaning desire for the given parameter to be decreased or increased, respectively. The second part of the questionnaire focused on garment elements worn by respondents, their recent physical activity, time spend outdoors and purpose of visit in that particular place. In the third part of the questionnaire, personal data such as gender, age, health status, country of origin and time of stay in Warsaw were collected. Exemplary weather perception questionnaire used in this study in transitional seasons is available online in Appendix 1. Total clothing insulation as well as particular clothing garment insulation was calculated according to ISO 9920 (2007).

#### Statistical analysis

In order to verify whether the purpose of staying outdoors influenced thermal perception, Student’s *t* test was used. To investigate relations between respondent thermal sensations and current biometeorological conditions (defined by PET and particular weather elements), regression analyses were applied. To analyse relationships between respondents’ personal features and their thermal sensation votes (TSV) or thermal preference votes (TPV), the C-Pearson’s contingency coefficient for nominal variables was used. All statistical calculations were made in IBM SPSS 22 software.

## Results

### Interviewees’ characteristics

**Fig. 2:**
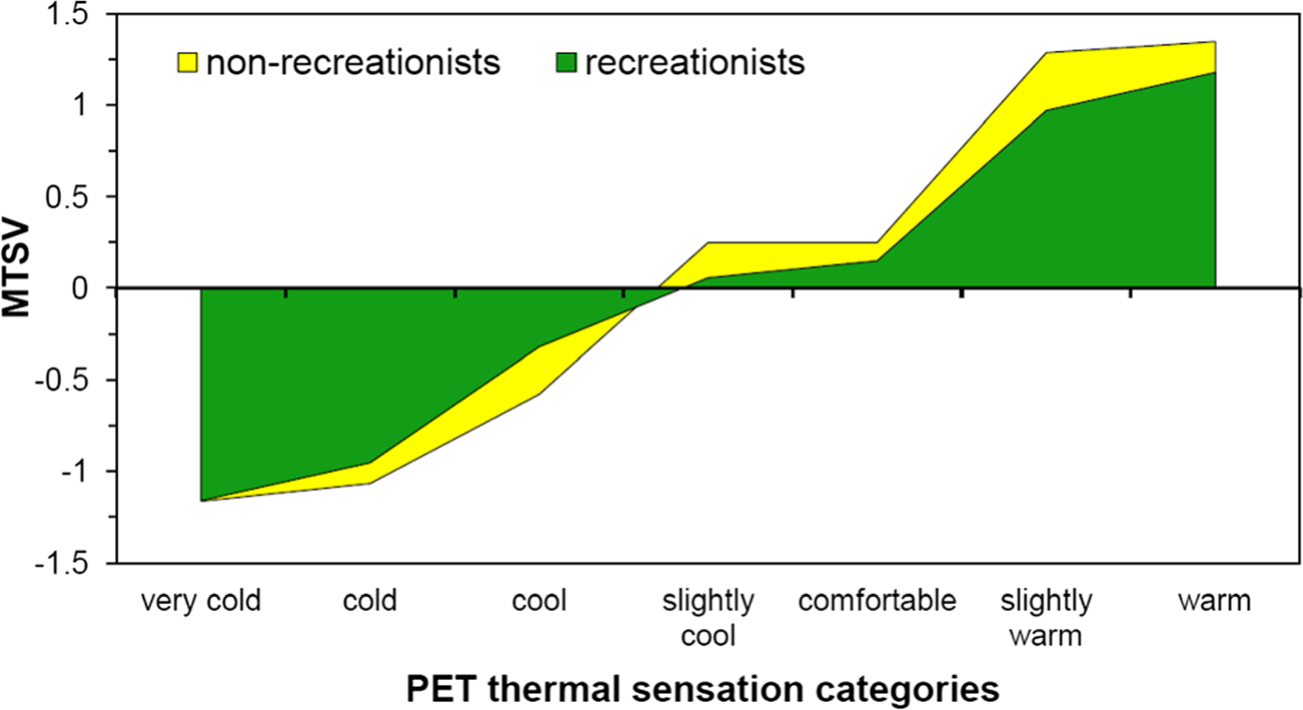
Recreationists’ and non-recreationists’ mean thermal sensation votes (MTSV) juxtaposed to *PET* thermal sensation ranges

During field studies in the Old Town Marketplace, a total of 818 questionnaires were collected. As some interviewees (156 persons) had come to the spot for other than tourist or recreational purposes, we first analysed whether this factor influenced their thermal perception. Mean thermal sensation votes (MTSV) were calculated for both recreationist and non-recreationist groups in PET thermal sensation ranges (Fig. 2). The thermal sensations of recreationists and non-recreationists differed significantly (*t* = −4.695; *p* = 0.002). People staying outdoors for tourism and recreation usually assessed thermal conditions in less extreme manner than the others, and their mean thermal sensation votes (MTSV) were always closer to the thermoneutral zone (0). Thus, non-recreationist group was excluded from the sample and 662 questionnaires were used in further analysis.

Among chosen 662 respondents, the disproportion between women and men number was small, 52.7 to 47.3 %, respectively. The most frequent age group was 15 to 29 years (55 %), followed by 30 to 44 years (26.7 %) and 45 to 65 years (15.4 %). The other age groups, which are usually ignored in thermal comfort studies (below 15 years and over 65 years), were represented by only 2.9 % of the sample. The largest share of respondents (77.2 %) had been walking at least for 15 min before the survey. Forty percent of respondents were Warsaw citizens staying at the Old Town for recreational purpose, while domestic tourists and visitors represented 36.3 % of the sample. Foreign tourists were the smallest group (23.7 %), and in majority, they originated from European countries (e.g. France, Spain, Germany). 39.6 % of interviewed people could be considered as acclimatized to local weather conditions as they had spent more than 7 preceding days in Warsaw or its close surroundings. 81.4 % of interviewees (mainly Poles) came from the hemiboreal warm summer continental climatic zone (Dfb). Among foreign tourists, 38.2 % originated from oceanic climatic zone (Cfb), followed by hemiboreal climatic zone (Dfb) (21.7 %), Mediterranean climates (Csa/Csb) (10.8 %) and hot summer continental climatic zone (Dfa) (9.4 %) inhabitants. Remaining climatic zones were represented by single individuals. 13.3 % of respondents reported chronic health issues (hypertension, coronary or rheumatic diseases, respiratory system disease and asthma etc.), but after confirming very weak and statistically insignificant (*p* > 0.05) relationship between health state and thermal sensations or preferences, we decided to include this group in the sample.

Clothing thermal insulation (Icl) is an important factor influencing thermal sensations of people, especially in continental climates, where air temperature fluctuates considerably during the year. In winter, clothing of 1.3–1.4 clo was used most frequently. In transitional seasons, when air temperature varied between 8 and 18 °C, Icl changed from nearly 1.3 to 0.9 clo, whereas on hot summer days, clothing that provided insulation of 0.4 clo was usually used.

### Biometeorological conditions in the study period

Prevailing weather conditions during the sampling periods were often typical for the seasons and for the central districts of Warsaw, except from July 2010, when a heat wave occurred (Table 1). During summer field survey, mean air temperature was 27.1 °C, with maximum values up to 30 °C. Mean global solar radiation ranged between 137.6 W·m^−2^ in autumn and 682.6 W m^−2^ in summer. High standard deviation values of Kglob indicated that insolation varied considerably in each season, due to dynamically changing cloudiness during the course of the day. In autumn and winter, relative humidity was 69 and 67 %, respectively, whereas in summer, it decreased to 37 %. In all seasons, very low wind speeds (<1 m·s^−1^) were registered, which was due to compact building downtown.

**Table 1:** Meteorological conditions during field surveys at the Old Town of Warsaw

Season	Air temperature (°C)				Global solar radiation (W·m^−2^)				Relative humidity (%)		Wind speed (m·s^−1^)	
	Average	SD	min	max	Average	SD	min	max	Average	SD	Average	SD
Spring	14.3	1.5	11.5	17.3	450.4	246.7	80.6	865.6	50	6	0.8	0.4
Summer	27.1	1.2	25.1	30.0	682.6	213.2	90.6	901.9	37	7	0.7	0.3
Autumn	9.8	0.7	8.5	11.8	137.6	120.6	14.4	569.4	69	10	0.7	0.4
Winter	−2.9	1.4	-6.7	-1.3	223.6	147.3	33.1	453.1	67	11	0.7	0.3

During field surveys in Warsaw, PET values changed throughout the year from −12.3 °C in January to 38.0 °C in July reflecting thermal sensations from very cold to hot (data not shown). In winter, very cold sensation dominated (96.3 %), while in summer, warm class prevailed (71.4 %) (Table 2). In spring, the PET index most frequently indicated cool sensation (38.1 %), followed by slightly cool (31.2 %) and neutral (25.7 %) ranges, whereas in autumn, cold (48.4 %) and cool (37.7 %) PET classes were observed most often. In October, in 5.7 % cases, warm PET category was observed, which was a consequence of extreme variations in cloudiness and solar radiation intensity at the time of the survey.

**Table 2:** Thermal sensation frequencies (%) according to PET values for Central Europe (Matzarakis and Mayer 1996)

PET categories	Spring (%)	Summer (%)	Autumn (%)	Winter (%)	Mean for all series (%)
Very cold (<4 °C)	0.0	0.0	0.0	96.3	23.7
Cold (4–7.9 °C)	2.0	0.0	48.4	0.0	9.5
Cool (8–12.9 °C)	38.1	0.0	37.7	3.7	19.5
Slightly cool (13–17.9 °C)	31.2	0.0	5.7	0.0	10.6
Neutral (18–22.9 °C)	25.7	4.0	0.8	0.0	9.1
Slightly warm (23–28.9 °C)	3.0	14.3	1.6	0.0	5.0
Warm (29–34.9.9 °C)	0.0	71.4	5.7	0.0	19.9
Hot (35–40.9 °C)	0.0	10.3	0.0	0.0	2.7

### Thermal sensations and preferences in different seasons

**Fig. 3:**
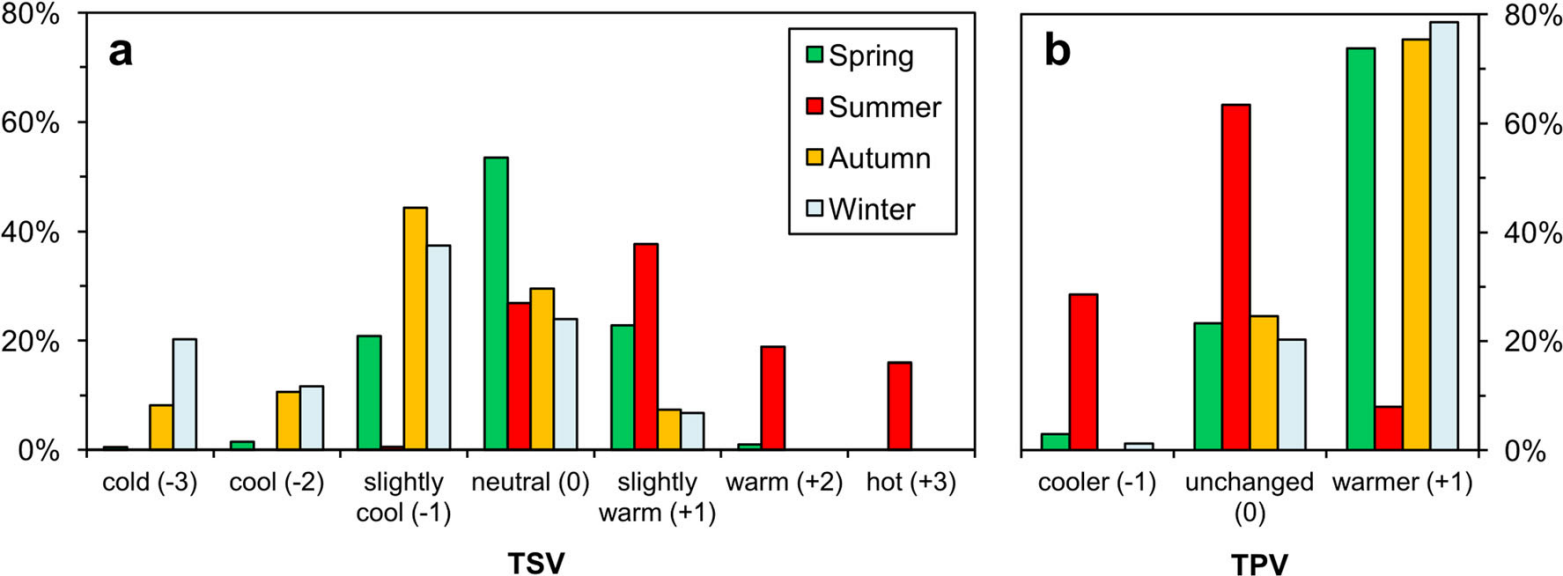
Frequency (%) distribution of respondent thermal sensation votes (TSV) (**a**) and thermal preferences votes (TPV) (**b**) in particular seasons (*N* = 662)

Thermal sensation votes (TSV) of respondents varied greatly during the year, but regardless of the season, most frequently the interviewees perceived thermal comfort or subcomfort, defined by ‘slightly cool’ (−1) or ‘slightly warm’ (+1) sensations (Fig. 3a). In winter and autumn, slightly cool sensations prevailed (37 and 44 % respectively), whereas in spring, maximal frequency of responses fell on neutral sensation (53 %). In the summertime, interviewees most frequently declared feeling slightly warm (38 %), although neutral thermal sensations were also very common (27 %). Regarding thermal preference votes (TPV), in winter and in transitional seasons, the majority of respondents preferred warmer thermal conditions with less than quarter interviewees expressing satisfaction with air temperature (Fig. 3b). On the other hand, in July, the majority of questioned people accepted thermal conditions and wished them not to change.

**Fig. 4:**
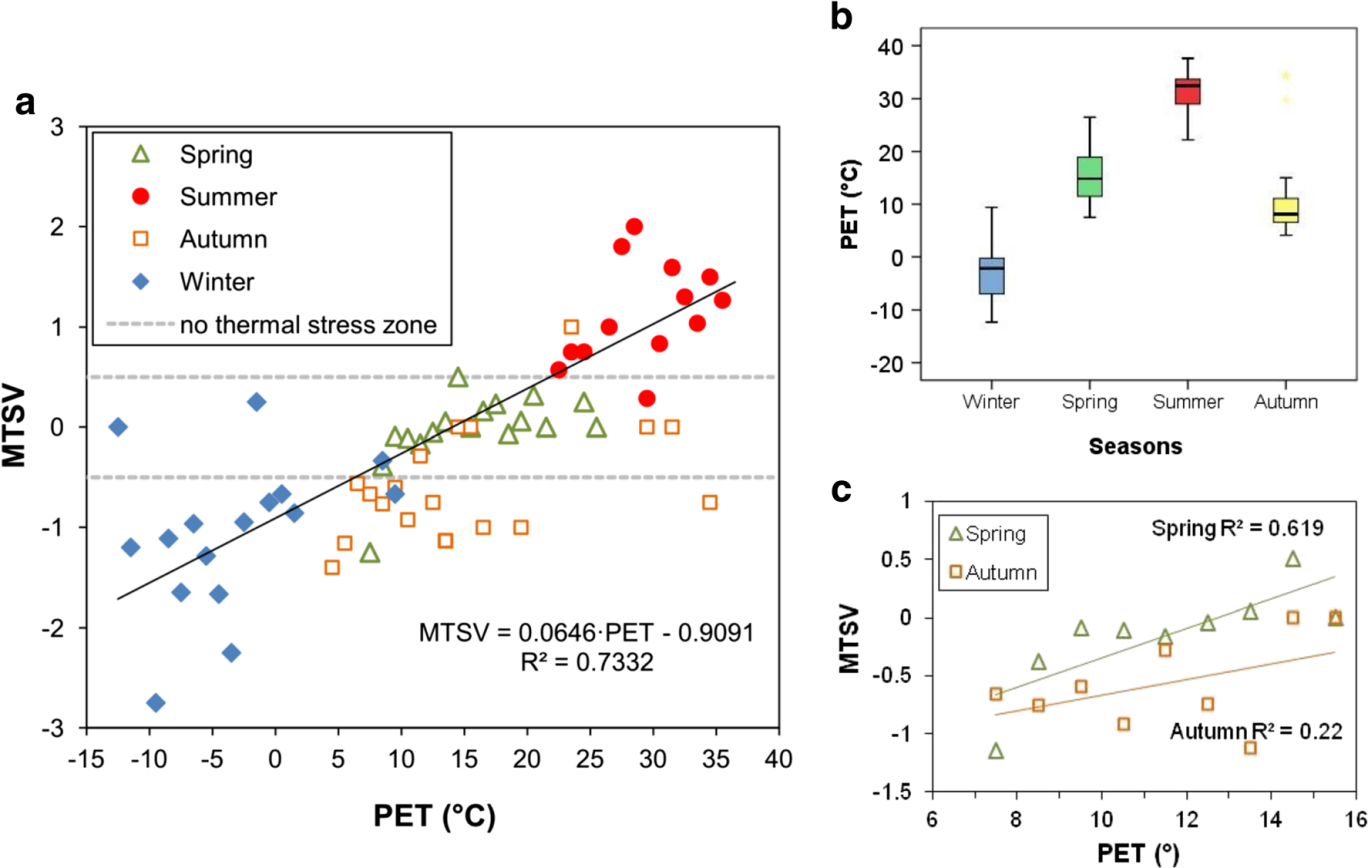
Mean thermal sensation votes (MTSV) versus PET values: in seasons (**a**), only in Spring and Autumn—common range (**c**). The extent of PET values regarded as neutral thermal sensations (TSV = 0) in particular seasons versus PET values (**b**)

Across the year, a linear relationship between biometeorological conditions defined by PET index and thermal sensations of the respondents was observed. After dividing PET values into 1 °C bins and calculating mean thermal sensation vote values, moderately strong correlation was found between MTSV and PET index (*R* ^2^ = 0.733, *p* < 0.0001) (Fig. 4a). Using linear trend equation, it was possible to determine PET values regarded by recreationists in Warsaw as neutral thermal zone. Basing on the assumption that −0.5 to +0.5 range of MTSV corresponds to ‘no thermal stress’ (Matzarakis et al. 1999), the neutral temperature, described as temperature at which people feel neither cool nor warm (Fanger 1972), was defined by PET values from 6.3 to 21.8 °C. However, when analysing only neutral sensation votes (TSV = 0; *N* = 230), different ranges of PET values can be observed for particular seasons (Fig. 4b). Median of PET for neutral thermal sensations varied from −2.1 °C in winter, 8.1 °C in autumn, 14.9 °C in spring and up to 32.4 °C in summer. Moreover, taking into account only common range of PET values in transitional seasons, TSV in spring were different than in autumn in comparable biothermal conditions. In spring, respondents generally declared feeling a little warmer than in autumn (Fig. 4c). These differences in thermal perception depending on short-term thermal history are known as perceptual alliesthesia effect, described by Spagnolo and de Dear (2003).

**Fig. 5:**
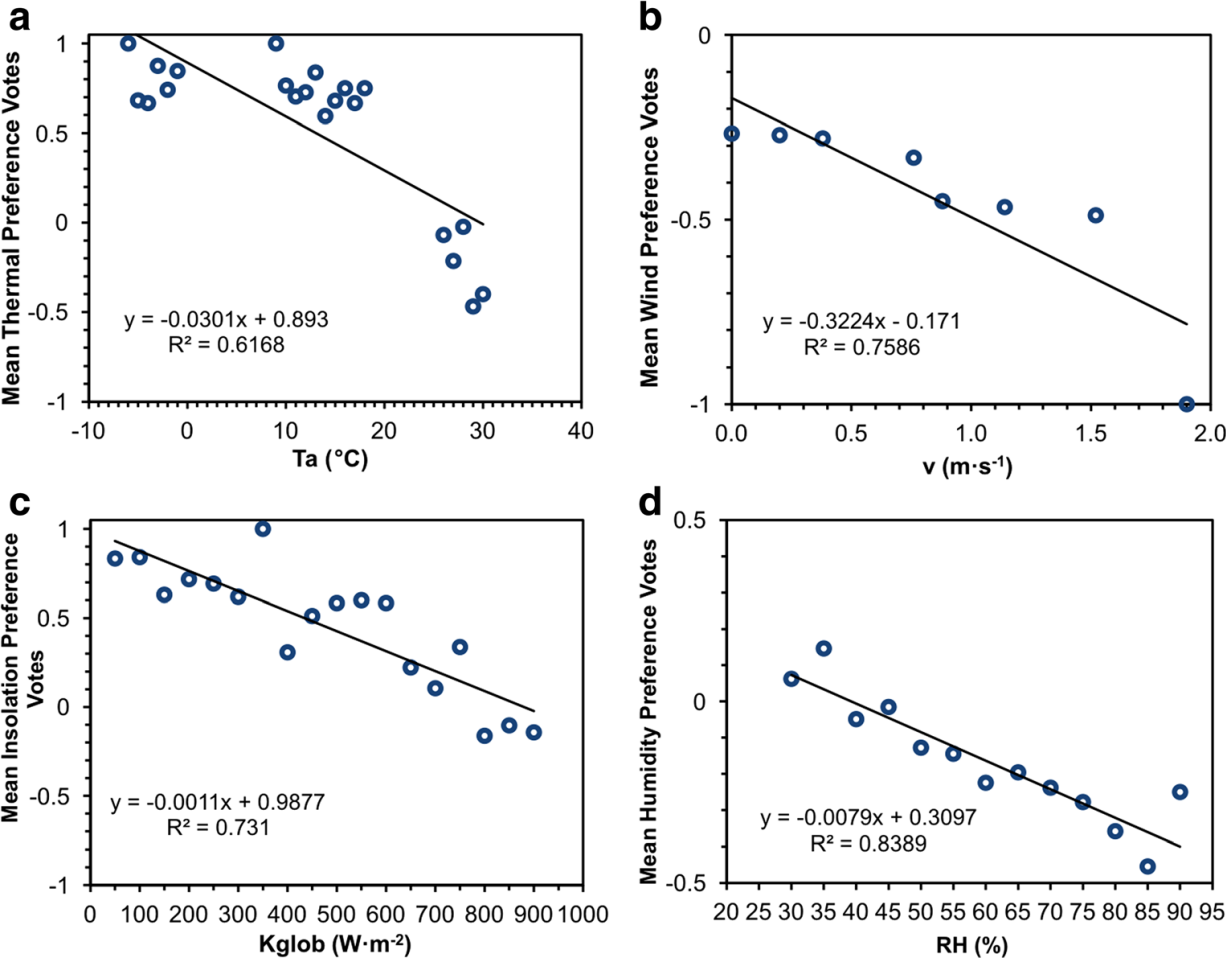
Mean preference votes for meteorological parameters: (**a**) air temperature, (**b**) wind speed, (**c**) global solar radiation, (**d**) relative humidity

Subsequently, mean preference votes (MPV) of the respondents were presented as a function of the corresponding meteorological elements (Fig. 5). With rising values of *Ta*, *v*, *Kglob* and *RH*, a preference for lower intensity of weather parameters was declared. These negative relationships were relatively strong, with *R* ^2^ > 0.6 and statistically significant at *p* < 0.0001, with the exception of wind speed which was significant at *p* < 0.01. From all of the analysed correlations, surprisingly, the strongest connection was found between *RH* and humidity preferences (*R* ^2^ = 0.839). When *RH* values raised to 39 %, the respondents tended towards less moisture in the air, although preferences for ‘no change’ in humidity prevailed in all seasons regardless of biothermal conditions. According to the fitted regression lines, interviewees preferred more intensive sunshine up to 898 W·m^−2^ and lower wind speed for the whole study period, irrespective of actual, very weak air movement (<2 m·s^−1^). The weakest relationship was noticed between air temperature and thermal preferences (*R* ^2^ = 0.617). Only when air temperature raised to 30 °C, respondents started to prefer cooler thermal conditions.

**Fig. 6:**
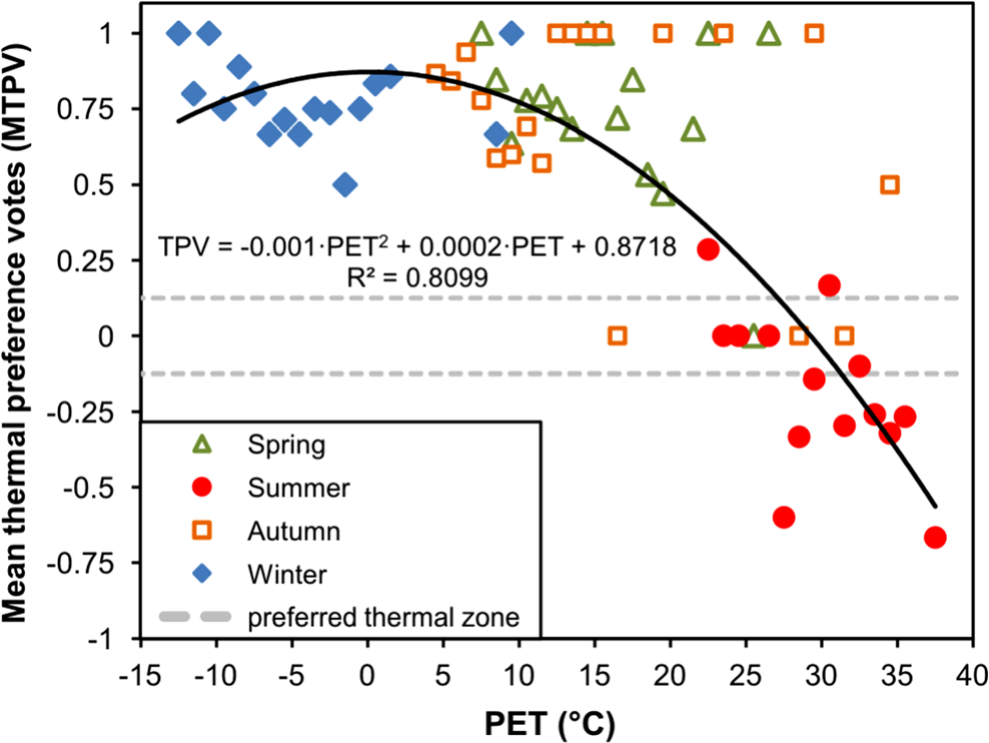
Mean thermal preference votes (MTPV) versus 1 °C PET values in seasons

To calculate optimal thermal zone for urban tourism and recreation during the year, binomial regression model for mean thermal preference votes (MTPV) in 1 °C PET ranges was used (Fig. 6). We applied procedure designed by Kovács et al. (2015), where the PET values corresponding to the ±0.125 interval of the MTPV are considered as comfortable. Using this approach, preferred spectrum of thermal conditions for tourism and recreation in Warsaw could be associated with PET range between 27.3 and 31.7 °C. These values represent upper border of slightly warm and warm thermal sensations based on the PET-scale for Central Europe and are much higher than thermoneutral range determined earlier.

**Fig. 7:**
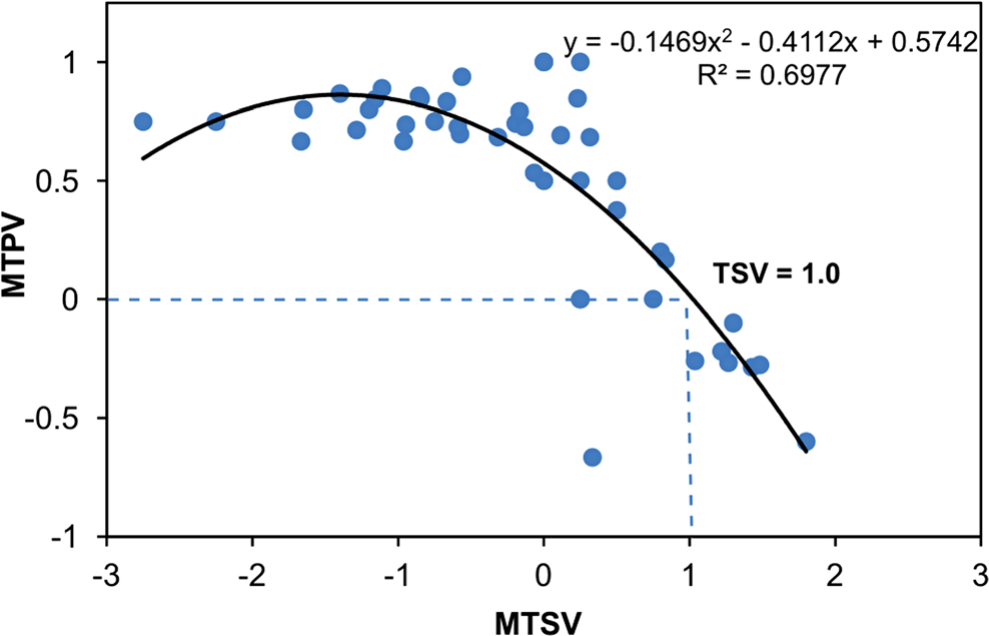
Respondents’ mean thermal preferences compared with corresponding mean thermal sensation votes in 1 °C PET ranges

Subsequently, we compared thermal sensations of the respondents with their thermal preferences. The Spearman’s correlation between TPV and TSV turned out to be negative and moderately strong for the whole year (*ρ* = −0.53, *N* = 662, *p* < 0.0001). When analysing MTPV of the respondents with their corresponding MTSV in adequate 1 °C PET ranges, in most cases prevailed preferences for slightly warmer than actual conditions (Fig. 7). For MTSV lower than or equal 0 (neutral), MTPV were usually between 0.5 and 1, which indicated wishes for higher than current air temperature. Assuming that MTPV = 0 is related with comfortable and desired thermal environment, the preferred thermal sensation for tourists and people staying outdoors for pleasure equals 1, which stands for ‘slightly warm’ sensation.

### Impact of personal factors on weather perception and thermal preferences

**Fig. 8:**
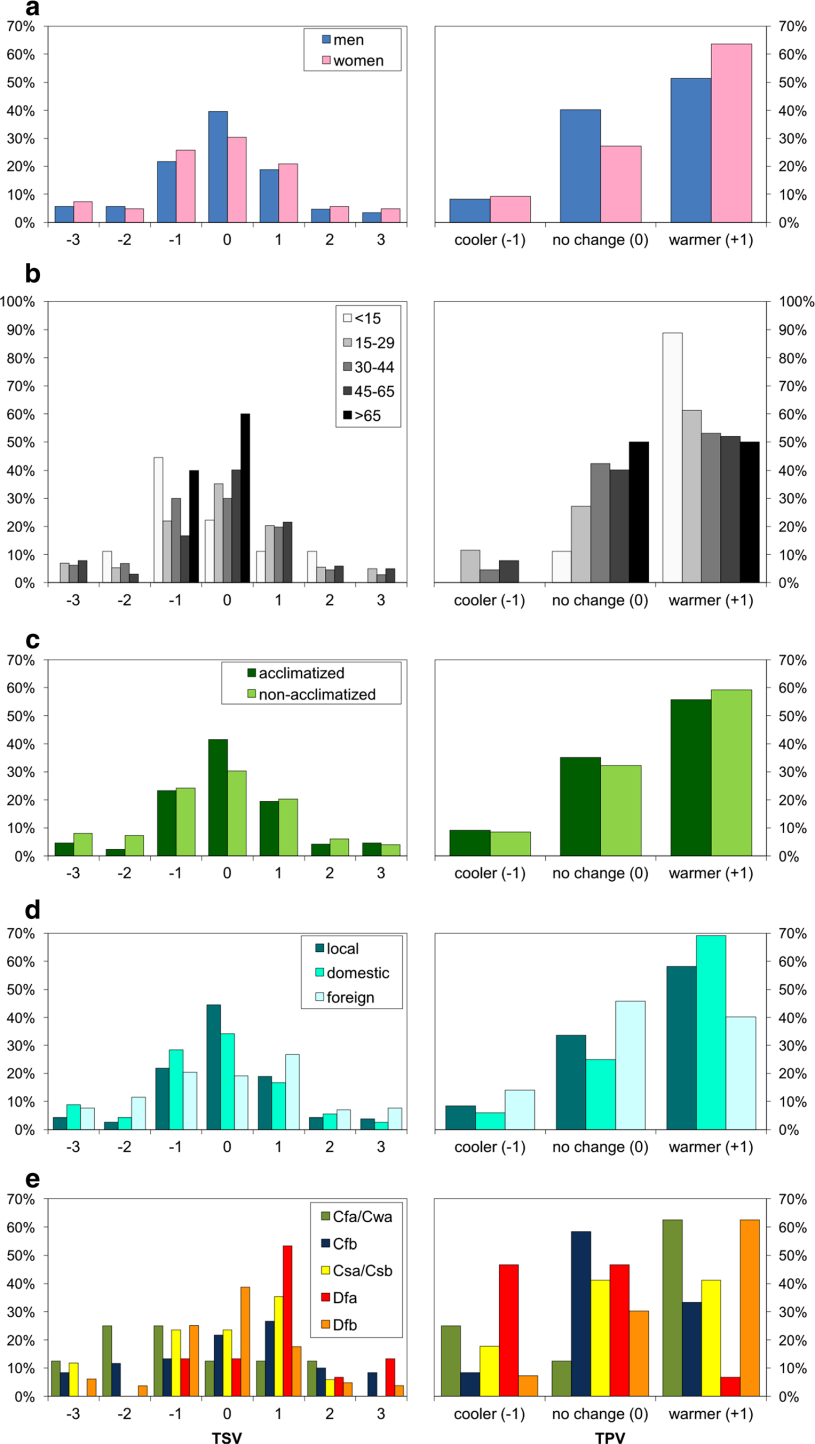
Frequency of thermal sensation votes (TSV) and thermal preference votes (TPV) depending on respondents’ personal features: (**a**) gender, (**b**) age, (**c**) acclimatization level, (**d**) origin, (**e**) provenance climatic zone TSV: *−3*—cold, *−2*—cool, *−1*—slightly cool, *0*—neutral, *+1*—slightly warm, *+2*—warm, *+3*—hot

In analysed group of tourists and people staying outdoors for recreation in Warsaw, men more frequently (9.2 pp) than women perceived thermal conditions as neutral (0), while women expressed higher susceptibility to deviations from thermal optimum and more frequently than men were dissatisfied with air temperature (Fig. 8a). However, the observed relationship was not statistically significant (contingency coefficient *C* = 0.106; *p* = 0.277). On the other hand, men more frequently (13 pp) than women wished no changes in their thermal environment, while women usually preferred warmer conditions (12.2 pp) (*C* = 0.138; *p* = 0.002). In the analysed sample, no statistically significant relation was found between respondents’ age and their thermal sensations (*C* = 0.183; *p* = 0.516), although the oldest group tended to choose most frequently neutral thermal sensation vote (Fig. 8b). Taking into account thermal preferences of the analysed sample, the acceptance of thermal conditions increased with age. Particularly, the older respondents less frequently wished warmer weather (*C* = 0.188; *p* = 0.002). It is generally accepted that level of acclimatization may have impact on human thermal perception. In our study, a person staying for at least 7 days in Warsaw or its surroundings was considered as acclimatized to local bioclimate. In analysed sample, acclimatized respondents more frequently perceived thermal conditions as neutral (11.4 pp) than non-acclimatized ones (Fig. 8c). The relationship between thermal sensation votes and acclimatization level was weak but significant (*C* = 0.159; *p* = 0.008), whereas no dependence was found between thermal preferences and adaptation to local bioclimate in Warsaw (*C* = 0.035; *p* = 0.668). However, when analysing respondent places of origin, local people (citizens of Warsaw and its surroundings) the most frequently (44.5 %) declared feeling neutral in current thermal conditions, while foreign tourist most often felt ‘slightly warm’ (26.8 %) (Fig. 8d) (*C* = 0.275, *p* < 0.0001). Concurrently, foreigners usually wished no changes in thermal environment (45.9 %), while Polish respondents significantly more frequently preferred the weather to be warmer (*C* = 0.22, *p* < 0.0001).

To our knowledge, the influence of visitor’s residency place climate on thermal perception has not been studied so far in urban environment. To address that, provenance climate of the respondents was characterized using Köppen Climate Classification. Climatic zones represented by less than five cases were excluded from further analysis. Cfa and Cwa types as well as Csa and Csb types were combined into subtropical and Mediterranean types, respectively, due to small representativeness of each group and existing similarities between them (Błażejczyk et al. 2015). People originating from the hemiboreal warm summer continental climatic zone (Dfb), the same climate as in Warsaw, most frequently perceived thermal conditions as neutral (38.8 %), while interviewees from hot summer continental climatic zone (Dfa) most frequently declared slightly warm sensations (53.3 %) (Fig. 8e). Respondents from temperate climates (Csa/Csb and Cfb) most often felt slightly warm during the year (35.3 and 26.7 %, respectively), although people from subtropical climates (Cfa/Cwa) most frequently characterized their sensations as cool and slightly cool (25 % each). People from Cfb climatic zone were most frequently satisfied with thermal conditions and wished it not to change (58.3 %), whereas respondents coming from Cfa/Cwa and Dfb climates most often preferred warmer weather (62.5 % each). The relationship between provenance climatic zone of the interviewees and their TSV as well as TPV was the strongest one out of all analysed factors (*C* = 0.281 and *C* = 0.285, respectively; *p* < 0.0001).

## Discussion and conclusions

The presented study was based on the assumption that people staying outdoors in the cities for tourism or recreation would perceive thermal environment in a different way than passers-by or people performing their work. Thermal perception of individuals cannot be entirely explained by the human energy balance, thus psychological and behavioural factors affect our thermal sensations and preferences towards weather elements (Spagnolo and de Dear 2003; Lin et al. 2011). People spending time outside the buildings of their own choice tend to accept current atmospheric conditions and willingly adapt to them in various ways (Nikolopoulou et al. 2001).

Outdoors, people have little control over biometeorological conditions in their surrounding; therefore, a freedom in choosing exposure for a particular weather reduces dissatisfaction with unfavourable thermal environment (Nikolopoulou and Steemers 2003; Nikolopoulou and Lykoudis 2006; Lin 2009). Moreover, while staying at particular place for relax, people are usually in a good mood and perceive thermal conditions as more comfortable (Knez and Thorsson 2008; Yin et al. 2012). Our findings show that due to voluntary nature of tourism and recreation, people staying outdoors for leisure tend to be more tolerant to various weather conditions and more often assess them as comfortable than those who are just passing by or staying at the spot out of necessity. This observation is consistent with the results of Thorsson et al. (2004).

We found a moderately strong relationship between MTSV of people staying for recreational purposes and biometeorological conditions defined by PET values. Thermal conditions regarded as neutral usually vary in different climatic zones and change throughout the year. Lin and Matzarakis (2008) found that occupants from tropical Taiwan regarded higher values of PET as neutral than occupants from Western/Middle Europe (26–30 °C versus 18–23 °C), whereas Cohen et al. (2012) specified neutral thermal zone for Mediterranean climate in Israel at 19–26 °C PET. In our study, neutral thermal conditions were determined using linear regression and delimited by −0.5 to +0.5 MTSV range. By applying this procedure, thermoneutral range was defined by PET values from 6.3 to 21.8 °C. Such a wide thermoneutral range is a consequence of two factors: (a) a large seasonal drift of biothermal conditions (broad PET amplitude during the year in Poland) and (b) small contribution of extreme thermal sensation votes (90 % of MTSV values fall between −1.5 and 1.5). The latter could be explained by clothing insulation adjustments and thermal adaptation of respondents throughout the year. Following the same methodology, Krüger et al. (2013) obtained a little narrower range (9–18 °C) of neutral conditions for warm half of the year in maritime moderate climate (Glasgow).

As frequently emphasized, neutral temperature values vary throughout the year (i.e. Nikolopoulou et al. 2001; Spagnolo and de Dear 2003; Nikolopoulou and Lykoudis 2006; Lin et al. 2011; Cohen et al. 2012). In accordance with this, in our study, median of PET for neutral thermal sensations (TSV = 0) varied from −2.1 °C in winter to 32.4 °C in summer. The changing with seasons weather perception could be, to some extent, associated with changing clothing insulation (Andrade et al. 2011; Lindner-Cendrowska and Błażejczyk 2013). Taking into account seasonal differentiation of thermal conditions in continental climatic zone, Kovács et al. (2015) suggested adjustments of neutral PET thresholds for each season separately, what according to our data, appears to be strongly justified in this type of climate. Moreover, our studies confirm the existence of alliestesia phenomenon, observed earlier by Spagnolo and de Dear (2003), Nikolopoulou and Lykoudis (2006) and Lin et al. (2011). In transitional seasons, respondents had different thermal sensation votes when experiencing identical thermal balance states, which was manifested in higher TSV values in spring than in autumn, under the same PET values.

Tourists, regardless of their origin, prefer sunny and warm weather at their holiday destination (Lise and Tol 2002), although various types of tourism activity are characterized by different preferences towards meteorological elements (Bafaluy et al. 2014). Consistently, our survey showed that in urban environment, people staying outdoors for tourism and recreation preferred warmer than actual thermal conditions for the majority of the year and even in summer, when PET exceeded 29 °C (warm thermal conditions), prevailed satisfaction with thermal environment. Kántor et al. (2012a) have claimed that humans are most vulnerable in their thermal perception to wind speed and insolation. In Warsaw, tourists in urban environment preferred intensive solar radiation and surprisingly weak wind speed (<2 m·s^−1^) throughout all seasons, what has not been observed elsewhere, but can be partially explained by small differentiation of wind conditions in dense built-up area of the Old Town of Warsaw.

We used binomial regression model to define optimal thermal range for tourism and recreation in urban areas in Poland. The obtained values of PET, between 27.3 and 31.7 °C, differ considerably from the limits proposed by Matzarakis and Mayer (1996) and are much higher than thermoneutral range obtained in our study. This preferred spectrum of thermal conditions, associated with slight and moderate heat stress, exceeds values identified for urban square users in Lisbon (21–23 °C PET) (Andrade et al. 2011), however is comparable with the set-point (29 °C PET) for Hungarian tourists in Szeged, above which prevails preference for cooler conditions (Kántor et al. 2012a). In addition, the comparison of TSV to TPV enabled us to confirm that people in Warsaw, during outdoor leisure activities, in general prefer to feel ‘slightly warm’ (TSV = 1). This finding is consistent with Humphreys and Hancock (2007) conclusions that comfortable thermal conditions are not identical with neutral and that in moderate climates, frequently thermal optimum is moved into the warm end of thermal sensation scale.

The influence of the selected personal characteristics on thermal perception has been examined in tourism climatology very rarely (Rutty and Scott 2015). Our results showing that women are more susceptible to deviations from thermal optimum, although with poor statistical significance, confirm conclusions of Oliveira and Andrade (2007). On the other hand, in our study, women significantly more frequently than men preferred air temperature to be higher, which can be explained by their higher emotional and physical sensitivity, as well as lower thermal insulation of clothing (Parsons 2002; Fato et al. 2004). This observation is opposite to the findings of Rutty and Scott (2015), whereby female beach users prefer to feel cooler than males. However, Bröde et al. (2012) or Yin et al. (2012) did not found influence of gender on thermal perception. Regarding age impact on thermal perception, we confirmed Unger et al. (2008), Andrade et al. (2011) as well as Krüger and Rossi (2011) conclusions that older people are less sensitive to temperature variations and less frequently prefer changes in their thermal environment, what can be to some extent a result of usually warmer clothing chosen by this age group. On the other hand, Rutty and Scott (2015) came to the opposite conclusions and observed that older beach users preferred warmer conditions than the younger ones. This conflicting results show that there are huge differences in weather perception between urban and beach tourists and that relations observed in particular sample cannot be applied to the other, involved in different type of leisure activity.

The place of origin has an impact on weather perception as it forms expectations of specific thermal conditions and therefore influences personal satisfaction from staying outdoors (Höppe 2002; Nikolopoulou and Steemers 2003). Foreign visitors usually have different expectations and thus different perception of tourist destination climate than local visitors (de Freitas 2003). In addition, Knez and Thorsson (2008) and Knez et al. (2009) proved that populations with divergent cultural backgrounds may vary in their thermal perceptions, even under similar biometeorological conditions. In our study, local respondents, with the best adaptation to Warsaw climate, most frequently perceived thermal environment as neutral, while foreign tourists often declared slightly warm thermal sensations. Taking into account that slightly warm conditions were considered optimal for urban tourism and recreation, it was logical that overseas visitors the least frequently wanted changes in their thermal environment. Rutty and Scott (2015) indicated that climatic region of origin can explain some differences in thermal perception of tourists from different countries, although very little attention has been given to this problem so far. In our study, subjects coming from hemiboreal climatic zone (Dfb), the same climate as in Warsaw, the most frequently declared feeling neutral, but at the same time usually wanted warmer thermal conditions. Tourists from subtropical climates (Cfa/Cwa) most often characterized their sensations as cool and slightly cool, concurrently preferring warmer weather. The penchant for higher air temperature, as well as cooler thermal sensations votes, is typical for residents of hot and humid regions (Lin 2009; Lin et al. 2011; Rutty and Scott 2015). Visitors from temperate climates (Csa/Csb and Cfb), characterized by higher air temperature (especially in winter), surprisingly most often felt slightly warm and were satisfied with thermal environment. The possible explanation is that they expected cooler weather in Poland and prepared themselves mentally and physically for less comfortable conditions. Obtained in advance knowledge about tourist destination’s climate could contribute to more positive weather assessment during holiday stay (Gómez-Martín 2005). Our novel findings concerning the influence of provenance climatic zone on thermal sensations and preferences need further studies, and it is highly recommended to include this aspect in future tourist thermal perception investigations.

## Electronic supplementary material

ESM 1 (PDF 555 kb)
